# Foetal Alcohol Spectrum Disorder and Offenders: Case Series

**DOI:** 10.1192/j.eurpsy.2026.11287

**Published:** 2026-07-31

**Authors:** K. Courtenay

**Affiliations:** Dept. of Psychiatry, UCL, London, United Kingdom

## Abstract

**Introduction:**

The impact of antenatal alcohol use may result in significant lifelong neurodevelopmental effects. As a spectrum disorder, foetal alcohol spectrum disorder (FASD) manifestation may vary widely with a range of effects in those affected. The prevalence of FASD in offenders is estimated from 1% to 5%. People with FASD are represented in offender services that are likely attributed to the behavioural and cognitive impairments associated with the disorder. Signs of autism and ADHD are seen in association with FASD.

**Objectives:**

To describe foetal alcohol spectrum disorder in forensic psychiatry services.

**Methods:**

The case series of two men illustrates the presentation of FASD in the criminal justice system. The purpose is to explore the presentation of FASD in forensic services and how it is detected and treated.

**Results:**

The two men were diagnosed in a forensic in-patient service where features were not detected in childhood. Their behaviour affected their education and life opportunities that led to offending due to the associated co-morbidities of FASD e.g. ADHD, social communication disorders, and learning problems. Stimulant medication has been effective in managing the ADHD signs of impulsivity, hyperactivity and inattention. Behavioural interventions related to appropriate communication and sex offender treatment have to take account of the cognitive impairments evident in both offenders.

**Conclusions:**

FASD is common and more prevalent in offender populations who are at greater risk of offending. Diagnosis of FASD is often made adulthood. Staff in Criminal Justice Services in general are not aware of the disorder. Screening for FASD is not commonly undertaken. More education on FASD for staff is required.

**Disclosure of Interest:**

None Declared

